# Exploring the relationship between housing conditions and risk perception in a disaster context

**DOI:** 10.1371/journal.pone.0310665

**Published:** 2025-10-15

**Authors:** Amer Hamad Issa Abukhalaf

**Affiliations:** Nieri Department of Construction and Real Estate Development, Clemson University, Clemson, South Carolina, United States of America; Arizona State University, UNITED STATES OF AMERICA

## Abstract

The relationship between housing conditions and risk perception is overlooked commonly in disaster studies. Correspondingly, this research study helps in filling this research gap by answering the two main following research questions, 1) Does individuals’ perception of hurricane risk vary based on their housing conditions?, and 2) Does this risk perception, in turn, influence their intention to take a hurricane protective action? For data collection, a quantitative approach was utilized, involving an online questionnaire that was filled by 816 subjects from five cities in Florida: Miami, Tallahassee, Jacksonville, Gainesville, and Ocala. In order to answer the first research question, many housing physical characteristics were statistically tested through variance analyses based on the survey responses collected; however, the only statistically significant variance found in risk perception among the survey subjects was based on two housing conditions; 1) Required Dwelling Repairs, & 2) If the Dwelling is on Ground-Floor or not. The variance had a medium strength for Threat Possibility, but was very weak for Threat Severity. Similarly, to answer the second research question, correlation and regression analysis were conducted to test the relationship between Threat Possibility and Threat Severity and the intention of preparing a supply emergency kit, an evacuation plan, and a communication plan. Risk perception had a weak correlation to the intentions of hurricane protective behaviors. Across all regression models, neither threat possibility nor threat severity showed statistically significant associations (p > 0.01) with preparedness intentions. By identifying specific housing conditions that influence risk perception, this research study has the potential to inform targeted interventions and educational campaigns to improve disaster preparedness among vulnerable populations. This can lead to better resource allocation and more effective community outreach programs. Moreover, the findings can guide policymakers and urban planners in designing and implementing building codes and housing regulations that enhance safety and resilience against hurricanes. This can result in improved living conditions and reduced vulnerability for residents in hurricane-prone areas.

## 1. Introduction

All storm preparedness actions fundamentally begin at home, as it represents the first line of defense against the impacts of any severe weather event. The household serves not only as a physical shelter but also as a psychological anchor during times of crisis. In the case of hurricanes, the most immediate and instinctive preparedness action is for individuals to remain indoors once the storm arrives. This precaution is so universally accepted that it often goes unstated. However, its importance cannot be overstated—no other preparedness measure can be effective if individuals are exposed to danger outside their homes during a hurricane’s landfall.

The perception of risk during such events is closely tied to how secure individuals feel within their own homes. This perception is shaped by both tangible and intangible factors, with the physical attributes of the dwelling playing a critical role. Elements such as the home’s location (e.g., coastal vs. inland), structural type (e.g., single-family home vs. apartment), construction materials (e.g., brick, wood, or concrete), presence or absence of a basement, and the size, type, and reinforcement of windows all contribute to a resident’s sense of safety.

Despite the clear relevance of these housing characteristics to hurricane risk perception, the relationship between the physical conditions of homes and how residents perceive and respond to storm risks remains significantly underexplored in disaster research [1–4]. To the best of our knowledge, no prior studies have conducted an in-depth investigation into the relationship between housing characteristics and risk perception, nor have they examined how this relationship may influence individuals’ intentions to engage in storm preparedness behaviors. While existing literature has explored various socio-economic and psychological factors affecting disaster readiness [5–8], the physical conditions of housing—arguably one of the most immediate and tangible influences on perceived safety—remain largely overlooked in the context of severe weather events such as hurricanes [9–12]. Addressing this oversight is critical, as understanding how the physical structure of a home shapes risk perception could inform more effective, household-level preparedness strategies and policies aimed at increasing community resilience.

This research aims to address this critical gap by examining how the built environment shapes both risk perception and preparedness intentions. Specifically, it seeks to answer two central research questions: 1) Does individuals’ perception of hurricane risk vary based on their housing conditions? 2) Does this risk perception, in turn, influence their intention to take a hurricane protective action? By exploring these questions, this study contributes to a more nuanced understanding of household-level disaster preparedness and offers insights that may inform more effective future policy, household-level preparedness strategies and policies aimed at increasing community resilience.

## 2. Literature review

As mentioned earlier, the relationship between housing conditions and risk perception remains an underexplored area in disaster research, particularly in the context of severe weather hazards such as hurricanes and tornadoes. While much of the existing literature focuses on socio-demographic or psychological variables, the physical attributes of the built environment—and how they shape individuals’ perceptions of risk—have received comparatively little attention. Bernhard Lindner is one of the few researchers that investigated the relationship between the built environment and risk perception in a hurricane context. Lindner used a supplemental approach that allows people to simulate hurricane scenarios and visualize the water depth using photographs of their neighborhoods. He argues that this approach can enhance awareness and risk perception [9]. Lindner’s research study shows that the majority of the public lacks the technical knowledge about hurricanes, including hurricane surge text advisories and standard hurricane storm surge maps, even in hurricane-prone areas such as Florida and Louisiana [9].

Lindner believed that showing the threats at a neighborhood scale discourages the high development of risk-prone areas and makes the risk more apparent. He focused on vulnerable groups, including the elderly, students, and non-English speakers, as he believed that they could benefit the most from this tool of visual representation of an approaching storm [9]. Lindner wanted to test this approach further, and he conducted another research study to better understand the role of efficacy in educating people about the risk of hurricanes in South Carolina using his tool of visual representation. Lindner collected his data through two anonymous surveys that were completed by 575 people [10]. Lindner found that 25% of the subjects would have reconsidered moving into their current house if they had known about the risk of hurricanes in that area [10]. These findings underscore the critical role of visual and localized communication tools in shaping public understanding of hurricane risks—especially among vulnerable populations. By translating abstract data into personally relevant and easily interpretable imagery, Lindner’s approach demonstrates how design and presentation can significantly impact risk perception and decision-making. This reinforces the importance of integrating visual and experiential methods into preparedness strategies, especially in areas where technical comprehension of hazards is limited.

The housing conditions for some demographic groups have made them the center of work for numerous disaster researchers [11–13]. For example, several researchers focused in their work on the vulnerability of mobile home occupants’ in the face of tornados and hurricanes. In 2016, a research study was conducted in South Carolina to reveal which factors influence evacuation planning and intentions, and to provide new insights into perspectives of preparedness and tornado protective actions [14]. A wide range of qualitative and quantitative data was utilized in the research study, and several variables concerned with the physical characteristics of mobile homes were tested. House size and wind-resistant features were found to contribute to subjects’ level of concern and evacuation intention [14]. Building on the findings of that research study and other similar studies, Margarethe Kusenbach conducted a qualitative research study in 2017 to explore the difference between risk perception and objective risk among mobile home residents in hurricane-prone areas. She interviewed over one hundred mobile home residents in South Florida, and she proposed a symbolic interactionism theoretical framework highlighting patterns of risk perceptions and evacuation decisions among study subjects [15]. These studies directly relate to our research questions by highlighting how housing conditions influence both risk perception and preparedness intentions. Mobile home residents, often in structurally vulnerable dwellings, perceive greater risk yet face significant barriers to acting on it—underscoring the complexity of the relationship between perceived risk and preparedness behavior. While some individuals recognize the danger, their inability to evacuate or lack of trust in warnings demonstrates that risk perception alone does not ensure preparedness.

In another research study, Stephen Strader et al. wanted to identify possible reasons for the low rates of tornado evacuation among mobile home residents in southeast states [16]. The results indicated that in addition to the fact that mobile home residents are more physically and socioeconomically vulnerable to severe weather hazards, they are also disproportionately less served by emergency services and potential sheltering locations. The authors also found that travel time and distances between shelters and mobile homes are strong factors in evacuations decisions and intentions [16]. Simultaneously, Stephen Strader et al. studied the Beauregard-Smith tornado event in Alabama. They conducted a research study to illustrate how tornado disasters unfold from a structural engineering perspective, focusing on manufactured homes [17]. The research study findings indicated that although the tornado forecasts were timely and accurate, 19 of manufactured homes residents still died as they didn’t feel the urge to evacuate even though they lacked proper ground anchoring [17]. These studies highlight how structural vulnerabilities in mobile homes, compounded by limited access to resources and emergency services, create a uniquely high-risk situation for residents during severe weather events. The interplay between physical housing deficiencies and social factors such as income, mobility, and access to information intensifies these risks. Importantly, even when accurate warnings are issued, evacuation decisions are often hindered by logistical constraints or a diminished perception of urgency.

Another research study was conducted to examine public attitudes and explore perceptions and factors that might influence response to tornado events. A qualitative approach was used in this research study, where 11 forecasters and 45 mobile home residents participated in semi-structured interviews [18]. The research study findings indicated that the lack of specificity in warnings and messaging to evoke a shelter-seeking behavior was a critical issue for forecasters. In addition, most subjects were aware of government guidelines for seeking safe shelters but still chose not to follow those [18]. In a similar research study, a team of researchers surveyed 257 manufactured housing residents in Mississippi and Alabama to research study their perceived tornado risk, protective actions, decision-making, and beliefs about structural integrity [19]. It was found that those who can evacuate often feel they have no need to do so. In contrast, those who want to evacuate as they recognize the potential danger of sheltering in their manufactured housing often lack the resources to carry out such sheltering plans [19]. While knowledge of official sheltering guidelines is relatively widespread, it does not consistently translate into protective behavior. These studies emphasize the importance of not only improving the clarity and urgency of emergency messaging but also addressing the underlying barriers that prevent effective response. Socioeconomic constraints, perceived structural safety, and a sense of fatalism or false security all play roles in shaping decisions.

Despite these valuable contributions, the intersection of housing characteristics and risk perception remains insufficiently addressed in the broader disaster preparedness literature. Few studies have systematically examined how specific features of the built environment shape individuals’ understanding of risk and their subsequent behavioral responses to severe weather threats. And even in those studies that do touch on housing conditions, the focus has been very limited—typically restricted to basic factors such as house size, type, and location—while overlooking a wider range of structural and contextual variables that may influence risk perception and preparedness behavior, and so, our study seeks to address that gap.

## 3. Materials and methods

For data collection, a quantitative approach was utilized, involving an online questionnaire that was filled by subjects at five cities in Florida: Miami, Tallahassee, Jacksonville, Gainesville, and Ocala. The choice was made to conduct the survey in an online format to achieve a higher response rate. These five cities were strategically selected for their geographic diversity across the state of Florida and their institutional relevance to the research. Miami is located in southeastern Florida along the Atlantic coast; Tallahassee, the state capital, lies in the Florida Panhandle in the northwestern region; Jacksonville is situated in the northeastern part of the state near the Atlantic coast; Gainesville is an inland city in north-central Florida; and Ocala is located in the central interior region of the state. Each location represents a distinct part of the state (southern, northern, eastern, central, and inland regions), allowing for a broader and more representative understanding of storm preparedness behaviors across different environments. Additionally, the presence of partner institutions in each city facilitated the distribution of the online questionnaire and streamlined data collection efforts through established academic and professional networks

### 3.1. Questionnaire design.

All questions in the questionnaire were short and direct. The questionnaire had three main sections; the storm preparedness section, the housing information section, and the demographics section. This research study looked at three main behaviors; preparing an emergency supply kit, preparing an evacuation plan, and preparing a communication plan. The questions about risk perception were general for all three behaviors; however, questions about the preparedness intention were specific for each behavior. Questionnaire Face Validity (QFV) was established by asking five researchers who understand the background of the research study, to read through the questionnaire in order to evaluate whether the questions capture the topic being investigated. Their comments were taken into consideration, and a modified version of the questionnaire was constructed accordingly. Then, the online questionnaire was pilot tested on 42 subjects. The subjects were asked to fill out the online questionnaire and give their feedback in order to make sure that the online version of the questionnaire was clear and free from any technical difficulties or typos and format un-clarity.

### 3.2. Data collection.

To ensure an adequate sample size, a confidence level of 95% and a confidence interval of 5 were taken into consideration in determining the sample size using the Cochran’s formula [20,21], and a minimum sample size of 384 subjects was determined. The online survey was sent out to possible subjects at the research study locations using the established academic and professional network of Florida Climate Institute (FCI), who agreed to help in the data collection for this research study. The survey recruitment period for the online survey was from 8/29/2022–12/12/2022.

### 3.3. Data analysis.

Each behavioral construct was measured through a set of questions in a Likert or Rising scales format. The answers were translated into percentages, for example, Strongly Disagree (0.0%), Disagree (25%), Neutral (50%), Agree (75%), and Strongly Agree (100%). The average percentage of the questions’ set for each construct was assigned as the construct’s final score, which was later used in the rest of the analysis. All behavioral constructs are metric/continuous data ranging from 0.0 to 1.0 (or 0% to 100%). The Cronbach’s Alpha (CA) was used here to check internal consistency for the responses gathered, with a minimum acceptable value of (0.75). Using Excel, DataTab, and SPSS software package, different statistical approaches were used, including Kruskal-Wallis, Mann-Whitney U-Test, Kendall’s Tau, Spearman correlations, and binary logistic regression, to uncover patterns and quantify variables through usable statistics based on generating numerical data, in a way that it can quantify behaviors and attitudes so that it can be generalized to the different groups in Florida [21]. For the logistic regression analysis, preparedness intentions were categorized as follows: scores below 40% (or 0.4) were classified as “No” or “Will Not Prepare,” scores between 40% and 60% (0.4–0.6) as “Indecisive,” and scores above 60% (or 0.6) as “Yes” or “Will Prepare.” In addition, we used the Variance Significance Factor (VSF) to address the dimensionality in the variance analysis for the data collected [22].

### 3.4. Ethics statement.

This study received ethical approval from the University of Florida Institutional Review Board (IRB), under reference number IRB202200544. Informed consent was obtained from all participants as part of the survey process, in the knowledge that no identifiable data was collected throughout the research study in order to protect the confidentiality of all subjects. The authors declare no competing interests and report that no specific funding was received to support this research.

## 4. Results

We received 816 survey responses. The survey sample was predominantly female (62.37%), with males comprising 35.39% and non-binary individuals 2.24%. In terms of race and ethnicity, the majority identified as White (53.22%), followed by Mixed Race (12.09%), Asian (10.12%), Latino (9.99%), Black (9.86%), and South Asian (4.73%). The majority had lived in hurricane-prone areas for over 10 years (60.81%), and respondents were nearly evenly split on whether they had previously experienced a hurricane. See Table 1 for subjects’ demographics.

**Table 1:** Sample demographics (N = 816).

Demographics	Frequency	
	n	%
Age		
19 or less	238	30.51%
20 - 24	170	21.79%
25 - 29	106	13.59%
30 - 34	65	8.33%
35 - 39	46	5.90%
40 - 44	36	4.62%
45 - 49	26	3.33%
50 - 54	24	3.08%
55 - 59	22	2.82%
60 - 64	22	2.82%
65+	25	3.21%
Total	780	100%
Gender Identity		
Female	474	62.37%
Male	269	35.39%
Non-binary	17	2.24%
Total	760	100%
Race/Ethnicity		
White	405	53.22%
Asian	77	10.12%
South Asian	36	4.73%
Latino	76	9.99%
Black	75	9.86%
Mixed Race	92	12.09%
Total	761	100%
Location		
Gainesville	568	69.61%
Miami	50	6.13%
Tallahassee	36	4.41%
Ocala	113	13.85%
Jacksonville	49	6%
Total	816	100%
Living in Hurricane Prone Area		
Less than 2 years	134	17.05%
2 - 5 years	96	12.21%
6 - 10 years	78	9.92%
More than 10 years	478	60.81%
Total	786	100%

Many respondents lived in single-family homes (38.75%) or apartments/condos (33.63%), with a smaller portion in dorms or mobile homes. Most homes were either built after 2000 (40.89%) or required minor repairs (49.03%). Nearly one-third had three bedrooms, and the most common household size was two people (26.12%), though 16% lived with 10 or more occupants. One-third of households had dependents. Most respondents lived on the first floor (57.98%) or top floor (56.07%). Concrete/steel was the most common exterior wall material (64.81%), and over half of window and door glass types were double glazed. See Table 2 for more details on subjects’ housing information.

**Table 2:** Sample housing information (N = 816).

Housing Information	Frequency	
	n	%
Dwelling Type		
Single-family home	303	38.75%
Apartment/Condo	263	33.63%
Townhouse or Duplex	61	7.80%
Fraternity/Sorority house or Dorm	121	15.47%
Mobile or Manufactured home	34	4.35%
Total	782	100%
Dwelling Age		
Built before 2000	228	37.44%
Significant renovation after 2000	132	21.67%
Built after 2000	249	40.89%
Total	609	100%
Required Dwelling Repairs		
No repairs	324	41.91%
Minor repair	379	49.03%
Significant repair	70	9.06%
Total	773	100%
Dwelling Number of Bedrooms		
0 or 1	52	6.70%
2	137	17.65%
3	234	30.15%
4	196	25.26%
5	32	4.12%
6+	125	16.11%
Total	776	100%
Dwelling Number of Occupants		
1	81	10.37%
2	204	26.12%
3	117	14.98%
4	165	21.13%
5	53	6.79%
6.0 - 9.0	36	4.61%
10+	125	16.01%
Total	781	100%
Are there any dependents living in the dwelling?		
Yes	260	33.33%
No	520	66.67%
Total	780	100%
Dwelling Floor		
1^st^	378	57.98%
2^nd^	125	19.17%
3^rd^	90	13.80%
4^th^	35	5.37%
5.0^th^ - 12.0^th^	24	3.68%
Total	652	100%
Is Dwelling on Top Floor?		
Yes	439	56.07%
Multiple Floors	130	16.60%
No	214	27.33%
Total	783	100%
Is Dwelling on Ground Floor?		
Yes	378	48.28%
Multiple Floors	130	16.60%
No	275	35.12%
Total	783	100%
Is Dwelling Subjected to Falling Objects?		
Yes	619	82.75%
No	129	17.25%
Total	748	100%
Is Building on High Ground?		
No	342	53.52%
Yes	297	46.48%
Total	639	100%
Is Dwelling surrounded by Taller Buildings?		
No	668	88.71%
Yes	85	11.29%
Total	753	100%
Material of Dwelling Exterior Walls		
Concrete/Steel	431	64.81%
Mixed Materials	81	12.18%
Wood	153	23.01%
Total	665	100%
Dwelling Window Frames		
Hung	369	47.49%
Sliding	85	10.94%
Picture	53	6.82%
Casement/Egress	26	3.35%
Awning	17	2.19%
Mixed Types	227	29.21%
Total	777	100%
Dwelling Window Glass Type		
Single glazed	231	46.29%
Double glazed	268	53.71%
Total	499	100%
Dwelling Exterior Glass Doors		
No doors	173	22.44%
French	51	6.61%
Traditional	211	27.37%
Sliding	151	19.58%
Mixed Types	185	23.99%
Total	771	100%
Dwelling Exterior Doors Glass Type		
Single glazed	164	44.09%
Double glazed	208	55.91%
Total	372	100%

### 4.1. Variance analysis.

The variance analysis for the data was conducted using nonparametric methods, such as Kruskal-Wallis and Mann-Whitney U-Test (see Tables 3 and 4). Many housing physical characteristics were tested in the analysis based on the survey responses collected; however, the only statistically significant variance found in risk perception among the survey subjects was based on two housing physical characteristics; 1) Required Dwelling Repairs, & 2) If the Dwelling is on Ground-Floor or not. That significant variance had a medium strength for Threat Possibility, but was very weak for Threat Severity (see Table 5).

**Table 3:** Non-parametric variance analysis (Threat Possibility).

Threat Possibility					
Demographics and Housing Information	Mean	SD	Variance Analysis		
			Kruskal-Wallis/Mann-Whitney U-Test (two-tailed)		
			P-value	Groups causing variance	P-value (Excluding the groups causing variance)
Age (in years)					
19 or less	0.47	0.17	0.004*	19 or less	0.369
20–24	0.54	0.17			
25 - 29	0.48	0.19			
30 - 34	0.53	0.16			
35 - 39	0.55	0.19			
40 - 44	0.53	0.16			
45 - 49	0.55	0.13			
50 - 54	0.5	0.16			
55 - 59	0.57	0.18			
60 - 64	0.53	0.21			
65+	0.51	0.17			
Total	0.51	0.17			
Gender Identity					
Female	0.52	0.17	0.44	–	–
Male	0.5	0.18			
Non-binary	0.48	0.13			
Total	0.51	0.17			
Race/Ethnicity					
White	0.5	0.17	0.042	–	–
Asian	0.51	0.2			
South Asian	0.55	0.18			
Latino	0.52	0.15			
Black	0.56	0.18			
Mixed Race	0.5	0.15			
Total	0.51	0.17			
Location					
Gainesville	0.51	0.17	<0.001*	-Miami -Ocala	0.088
Miami	0.61	0.17			
Tallahassee	0.55	0.14			
Ocala	0.43	0.15			
Jacksonville	0.55	0.19			
Total	0.51	0.17			
Living in Hurricane Prone Area					
Less than 2 years	0.49	0.19	0.032	–	–
2 - 5 years	0.48	0.18			
6 - 10 years	0.48	0.17			
More than 10 years	0.52	0.17			
Total	0.51	0.18			
Dwelling Type					
Single-family home	0.5	0.17	0.067	–	–
Apartment/Condo	0.52	0.19			
Townhouse or Duplex	0.55	0.16			
Fraternity/Sorority house or Dorm	0.49	0.16			
Mobile or Manufactured home	0.46	0.14			
Total	0.51	0.17			
Dwelling Age					
Built before 2000	0.53	0.17	0.054	–	–
Significant renovation after 2000	0.49	0.17			
Built after 2000	0.5	0.17			
Total	0.51	0.17			
Required Dwelling Repairs					
No repairs	0.48	0.17	0.001*	No repairs	0.139
Minor repair	0.52	0.17			
Significant repair	0.55	0.19			
Total	0.51	0.17			
Dwelling Number of Bedrooms					
0 or 1	0.49	0.17	0.191	–	–
2	0.52	0.19			
3	0.49	0.17			
4	0.53	0.18			
5	0.51	0.15			
6+	0.49	0.17			
Total	0.51	0.17			
Dwelling Number of Occupants					
1	0.5	0.19	0.192	–	–
2	0.52	0.17			
3	0.48	0.17			
4	0.52	0.18			
5	0.48	0.16			
6.0 - 9.0	0.51	0.19			
10+	0.49	0.17			
Total	0.51	0.17			
Dependents					
Yes	0.51	0.18	0.93	–	–
No	0.51	0.17			
Total	0.51	0.17			
Dwelling Floor					
1	0.51	0.18	0.049	–	–
2	0.52	0.18			
3	0.49	0.16			
4	0.45	0.17			
5.0 - 12.0	0.44	0.16			
Total	0.5	0.18			
Is Dwelling on Top Floor?					
Yes	0.5	0.17	0.086	–	–
Multiple Floors	0.53	0.17			
No	0.51	0.19			
Total	0.51	0.18			
Is Dwelling on Ground Floor?					
Yes	0.51	0.18	0.058	–	–
Multiple Floors	0.53	0.17			
No	0.49	0.17			
Total	0.51	0.18			
Is Dwelling Subjected to Falling Objects?					
Yes	0.52	0.17	0.07	–	–
No	0.49	0.17			
Total	0.51	0.17			
Is Building on High Ground?					
No	0.53	0.18	0.006*	Yes	1
Yes	0.49	0.16			
Total	0.51	0.17			
Is Dwelling surrounded by Taller Buildings?					
No	0.5	0.17	0.069	–	–
Yes	0.54	0.16			
Total	0.51	0.17			
Material of Dwelling Exterior Walls					
Concrete/Steel	0.52	0.17	0.356	–	–
Mixed Materials	0.49	0.15			
Wood	0.51	0.18			
Total	0.51	0.17			
Dwelling Window Frames					
Hung	0.51	0.18	0.241	–	–
Sliding	0.55	0.19			
Picture	0.49	0.15			
Casement/Egress	0.52	0.17			
Awning	0.47	0.17			
Mixed Types	0.5	0.16			
Total	0.51	0.17			
Dwelling Window Glass Type					
Single glazed	0.53	0.18	0.244	–	–
Double glazed	0.51	0.18			
Total	0.52	0.18			
Dwelling Exterior Glass Doors					
No doors	0.51	0.17	0.142	–	–
French	0.57	0.18			
Traditional	0.5	0.17			
Sliding	0.51	0.19			
Mixed Types	0.5	0.16			
Total	0.51	0.17			
Dwelling Exterior Doors Glass Type					
Single glazed	0.52	0.18	0.582	–	–
Double glazed	0.52	0.17			
Total	0.52	0.18			

* p < 0.01.

Through a trial-and-error approach, we identified the specific group contributing to the observed variance for each independent variable. This was determined by systematically excluding one group at a time from the analysis and observing the change in the p-value. The group whose exclusion resulted in the p-value increasing to ≥ 0.01 was considered the primary driver of the original statistically significant variance.

**Table 4:** Non-parametric variance analysis (Threat Severity).

Threat Severity					
Demographics and Housing Information	Mean	SD	Variance Analysis		
			Kruskal-Wallis/ Mann-Whitney U-Test (two-tailed)		
			P-value	Groups causing variance	P-value (Excluding the groups causing variance)
Age (in years)					
19 or less	0.48	0.15	<0.001*	19 or less	0.043
20 - 24	0.54	0.15			
25 - 29	0.52	0.17			
30 - 34	0.56	0.13			
35 - 39	0.58	0.17			
40–44	0.6	0.15			
45 - 49	0.61	0.14			
50 - 54	0.58	0.18			
55–59	0.65	0.17			
60 - 64	0.59	0.22			
65+	0.54	0.17			
Total	0.54	0.16			
Gender Identity					
Female	0.54	0.16	0.623	–	–
Male	0.53	0.16			
Non-binary	0.53	0.14			
Total	0.54	0.16			
Race/Ethnicity					
White	0.54	0.16	0.567	–	–
Asian	0.53	0.18			
South Asian	0.55	0.18			
Latino	0.51	0.13			
Black	0.55	0.18			
Mixed Race	0.51	0.14			
Total	0.54	0.16			
Location					
Gainesville	0.55	0.16	<0.001*	- Miami - Ocala	0.179
Miami	0.63	0.16			
Tallahassee	0.59	0.13			
Ocala	0.44	0.14			
Jacksonville	0.53	0.19			
Total	0.54	0.16			
Living in Hurricane Prone Area					
Less than 2 years	0.52	0.16	0.118	–	–
2 - 5 years	0.5	0.17			
6 - 10 years	0.53	0.16			
More than 10 years	0.55	0.16			
Total	0.54	0.16			
Dwelling Type					
Single-family home	0.54	0.16	0.231	–	–
Apartment/Condo	0.55	0.17			
Townhouse or Duplex	0.55	0.16			
Fraternity/Sorority house or Dorm	0.51	0.14			
Mobile or Manufactured home	0.55	0.17			
Total	0.54	0.16			
Dwelling Age					
Built before 2000	0.57	0.16	0.025	–	–
Significant renovation after 2000	0.54	0.15			
Built after 2000	0.53	0.16			
Total	0.55	0.16			
Required Dwelling Repairs					
No repairs	0.51	0.17	<0.001*	No repairs	0.3
Minor repair	0.55	0.15			
Significant repair	0.59	0.17			
Total	0.54	0.16			
Dwelling Number of Bedrooms					
0 or 1	0.52	0.14	0.159	–	–
2	0.54	0.16			
3	0.53	0.16			
4	0.56	0.17			
5	0.53	0.16			
6+	0.51	0.15			
Total	0.54	0.16			
Dwelling Number of Occupants					
1	0.55	0.17	0.137	–	–
2	0.56	0.16			
3	0.52	0.16			
4	0.54	0.17			
5	0.53	0.15			
6.0 - 9.0	0.5	0.16			
10+	0.51	0.14			
Total	0.54	0.16			
Dependents					
Yes	0.53	0.18	0.336	–	–
No	0.54	0.15			
Total	0.54	0.16			
Dwelling Floor					
1	0.54	0.17	0.181	–	–
2	0.54	0.15			
3	0.53	0.16			
4	0.5	0.15			
5.0 - 12.0	0.47	0.11			
Total	0.53	0.16			
Is Dwelling on Top Floor?					
Yes	0.54	0.16	0.048	–	–
Multiple Floors	0.56	0.17			
No	0.52	0.17			
Total	0.54	0.16			
Is Dwelling on Ground Floor?					
Yes	0.54	0.17	0.074	–	–
Multiple Floors	0.56	0.17			
No	0.53	0.15			
Total	0.54	0.16			
Is Dwelling Subjected to Falling Objects?					
Yes	0.55	0.16	0.018	–	–
No	0.51	0.15			
Total	0.54	0.16			
Is Building on High Ground?					
No	0.54	0.17	0.776	–	–
Yes	0.54	0.15			
Total	0.54	0.16			
Is Dwelling surrounded by Taller Buildings?					
No	0.54	0.16	0.107	–	–
Yes	0.56	0.15			
Total	0.54	0.16			
Material of Dwelling Exterior Walls					
Concrete/Steel	0.54	0.17	0.654	–	–
Mixed Materials	0.54	0.13			
Wood	0.55	0.16			
Total	0.54	0.16			
Dwelling Window Frames					
Hung	0.53	0.16	0.138	–	–
Sliding	0.58	0.18			
Picture	0.5	0.12			
Casement/Egress	0.58	0.17			
Awning	0.56	0.16			
Mixed Types	0.53	0.16			
Total	0.54	0.16			
Dwelling Window Glass Type					
Single glazed	0.56	0.16	0.244	–	–
Double glazed	0.55	0.16			
Total	0.55	0.16			
Dwelling Exterior Glass Doors					
No doors	0.54	0.16	0.294	–	–
French	0.58	0.17			
Traditional	0.53	0.15			
Sliding	0.53	0.18			
Mixed Types	0.54	0.16			
Total	0.54	0.16			
Dwelling Exterior Doors Glass Type					
Single glazed	0.55	0.16	0.99	–	–
Double glazed	0.56	0.16			
Total	0.56	0.16			

*p < 0.01

Through a trial-and-error approach, we identified the specific group contributing to the observed variance for each independent variable. This was determined by systematically excluding one group at a time from the analysis and observing the change in the p-value. The group whose exclusion resulted in the p-value increasing to ≥ 0.01 was considered the primary driver of the original statistically significant variance.

**Table 5:** Variance Significance Factor (VSF).

#	Independent variable	Threat Possibility	Threat Severity
		VSF	VSF
1	Age (in years)	0.38	0.50
2	Location	0.75	0.63
3	Required Dwelling Repairs	0.50	0.01
4	Is Dwelling on Ground Floor?	0.38	N/A

VSF (Variance Significance Factor): Very weak (0,0 < 0,1), Weak (0,1 < 0,3), Medium (0,3 < 0,5), Strong (0,5 < 0,7), Very strong (0,7 < 1).

VSF is only calculated for the variables with a p < 0.01 (Kruskal-Wallis/ Mann-Whitney U-Test).

VSF is a value between 1 and 0, where 1 means that the variance significantly affects the other significant variances and isn’t being significantly affected by any of them, and 0 means that the variance doesn’t significantly affect the other significant variances, but it is being significantly affected by all of them.

### 3.2. Correlation and regression analysis.

We used Spearman’s correlation to test the relationship between the behavioral constructs for each protective behavior, and we tested the correlation between the intentions of the three protective behaviors. Even though the correlations were statically significant, the risk perception had a weak correlation to the intentions of hurricane personal protective behaviors (see Table 6). On the other hand, we found that the correlation between preparing an evacuation plan and preparing a communication plan is strong compared to the correlation of these two behaviors and preparing an emergency supply kit (see Table 6).

**Table 6:** Correlation and significance.

Variable		1	2	3	4	5
1.Threat Possibility	Correlation (r)	1				
	p					
2.Threat Severity	Correlation (r)	0.76	1			
	p	<.001				
3.Intention (Emergency Kit)	Correlation (r)	0.13	0.16	1		
	p	<.001	<.001			
4.Intention (Evacuation Plan)	Correlation (r)	0.29	0.28	0.55	1	
	p	<.001	<.001	<.001		
5.Intention (Communication Plan)	Correlation (r)	0.14	0.13	0.55	0.75	1
	p	<.001	<.001	<.001	<.001	

(r)-value: Very weak (0,0 < 0,1), Weak (0,1 < 0,3), Medium (0,3 < 0,5), Strong (0,5 < 0,7), Very strong (0,7 < 1).

Insignificant correlation (p-value > 0.01).

Logistic regression analysis was conducted to examine whether perceived threat possibility and severity predict intentions to prepare for three protective behaviors: an emergency supply kit, an evacuation plan, and a communication plan. Across all models, neither threat possibility nor threat severity showed statistically significant associations (p > 0.01) with preparedness intentions. Although some odds ratios were greater than 1—suggesting a positive relationship (e.g., OR = 4.84 for threat severity and supply kit intention)—the wide confidence intervals and high p-values indicate a lack of reliable predictive power (see Table 7).

**Table 7:** Logistic regression.

1.Intention of preparing an Emergency Supply Kit (Dependent Variable)						
Independent Variable	Coefficient B	Standard error	z	p	Odds Ratio	95% conf. interval
Threat Possibility	−0.12	1.13	0.1	0.916	0.89	0.1 - 8.1
Threat Severity	1.58	1.24	1.27	0.203	4.84	0.43 - 54.87
2.Intention of preparing an Evacuation Plan (Dependent Variable)						
Independent Variable	Coefficient B	Standard error	z	p	Odds Ratio	95% conf. interval
Threat Possibility	0.8	1.07	0.75	0.455	2.23	0.27 - 18.23
Threat Severity	0.62	1.12	0.55	0.582	1.85	0.21 - 16.74
3.Intention of preparing a Communication Plan (Dependent Variable)						
Independent Variable	Coefficient B	Standard error	z	p	Odds Ratio	95% conf. interval
Threat Possibility	0.75	1	0.75	0.455	2.11	0.3 - 14.97
Threat Severity	0.19	1.07	0.17	0.863	1.2	0.15 - 9.82

Insignificant correlation (p-value > 0.01).

## 5. Discussion

Threat possibility and threat severity together form the overall perceived risk perception [22–25]. The first research question was “Does individuals’ perception of hurricane risk vary based on their housing conditions?” In order to answer this research question, many housing physical characteristics were tested in the analysis based on the survey responses collected; however, the only statistically significant variance found in risk perception among the survey subjects was based on two housing physical characteristics; 1) Required Dwelling Repairs, & 2) If the Dwelling is on Ground-Floor or not. That significant variance had a medium strength for Threat Possibility, but was very weak for Threat Severity (see Table 5). This suggests that while certain tangible aspects of the home environment may influence how individuals assess hurricane risk, the overall impact is limited. This nuance challenges prevailing narratives in disaster preparedness literature and highlights the importance of targeting specific housing vulnerabilities rather than applying broad assumptions about housing and risk perception. Overall, the expectation was to see more variance in risk perception based on the different housing physical characteristics, but the analysis shows otherwise. This could explain why it’s hard to find any studies that looked in-depth into the impact of housing physical characteristics on hurricane risk perception; it is possible that the findings in other studies with regard to this matter were never reported as the relationship between the two was insignificant.

On the other hand, the second research question was “Does this risk perception, in turn, influence their intention to take a hurricane protective action?” To answer that question, correlation and logistic regression analyses were conducted to test the relationship between Threat Possibility and Threat Severity and the intention of preparing a supply emergency kit, an evacuation plan, and a communication plan. Risk perception had a weak correlation to the intentions of hurricane personal protective behaviors based on Spearman’s correlation analysis (see Table 6). In addition, logistic regression showed that perceived threat possibility and severity were not statistically significant predictors of intentions to prepare for supply kits, evacuation plans, or communication plans, as indicated by high p-values and wide confidence intervals (see Table 7). This didn’t come as a surprise since many researchers have very different opinions about the role that risk perception plays in disaster preparedness [26–30].

There have been many studies that looked into the role of risk perception in disaster preparedness. For example, a research study that was conducted in Romania showed a strong correlation between risk perception and evacuation plans [31]. A similar research study was conducted in Italy also showed that disaster preparedness was positively associated with risk perception [32]. Another two studies in Holland and Germany showed a significant positive correlation between risk perception and preparedness intention [33]. On the other hand, many studies showed that risk perception doesn’t have a significant impact on preparedness intentions. For example, a research study that was conducted in the 1980s showed that there is no significant correlation between perception of risk probability and people’s preparedness intentions for disasters in US and Canadian coastal cities [34]. And even more recent studies came to that same conclusion. A research study that was conducted in the US showed that perceived risk wasn’t significantly correlated with preparedness intentions or actual preparedness [35]. The same conclusion was found in another research study that looked into disaster preparedness in many countries around the world [36].

The question here is “Does risk perception play a role in disaster preparedness or not?” The answer to this question is that there is no easy answer to that; each case is different and it is really hard to generalize whether risk perception influences people’s intentions to prepare or not for natural hazards. For example, in our research study, risk perception had an insignificant impact on people’s intentions to prepare for the hurricane season (see Tables 6 and 7). Our data was collected during the hurricane season but there wasn’t an approaching storm; however, if there was an approaching storm, the risk perception could have had a significant impact on people’s intention to prepare for that particular storm. Consequently, if we want to motivate people to prepare for the hurricane season at the beginning of the season and without having any detected storm on the radar, the emergency communication shouldn’t be significantly highlighting the danger that a storm can cause or triggering a sense of danger among people because their risk perception doesn’t have a significant impact on their intentions to prepare at the point. At the same time, if there is a storm approaching, emergency communication needs to change and consider risk perception in the communication. Either way, using the same unified emergency communication for the entire hurricane season won’t be as effective because people perceive the same communication differently based on whether there is a storm approaching or not.

In addition, we found that the correlation between preparing an evacuation plan and preparing a communication plan is strong compared to the correlation of these two behaviors and preparing an emergency supply kit (see Table 6). This could mean that if someone chooses to prepare an evacuation plan, they are more likely to prepare a communication plan at the same time. These two behaviors are closer in nature to each other compared to preparing a supply kit. Preparing evacuation and communication plans is more fluid and requires more cognitive effort than preparing an emergency supply kit, especially that the instructions to prepare a supply kit are generally more straightforward and easier to follow and apply. In addition, preparing evacuation and communication plans does not require spending any money on buying certain items and does not require a space to store these items and does not require any intense physical activity like carrying heavy water bottles from the grocery store, which can make people look and perceive these behaviors differently.

These findings offer several practical applications for emergency management professionals, public policy makers, and community preparedness campaigns. First, since the physical characteristics of housing—particularly the need for repairs and ground-floor location—had some influence on risk perception, targeted interventions could be developed for residents in more vulnerable housing conditions. For example, offering free or subsidized home inspections or repair assistance to residents in older or poorly maintained homes may not only improve safety but also increase awareness and potentially enhancing preparedness behaviors over time. Second, because perceived risk did not significantly influence preparedness intentions outside of an imminent storm event, emergency communication strategies should be dynamic and timed strategically. During the early or calm parts of the hurricane season, messages should focus on convenience, efficiency, and cost-free preparedness actions, rather than emphasizing risk alone. Framing preparedness as a manageable and logical step—rather than a reaction to fear—may resonate more effectively with the public when risk perception is low. Conversely, when a storm is approaching and risk perception naturally rises, messaging should pivot to emphasize immediacy and threat, aligning with the public’s heightened concern. This dual-mode communication strategy could improve engagement and behavioral response across different stages of the hurricane season.

Moreover, because individuals who prepare evacuation and communication plans tend to do so in tandem, emergency programs can bundle these behaviors into a single outreach effort. Toolkits or digital platforms could offer step-by-step guidance on both plans simultaneously, reducing cognitive load and improving uptake. Meanwhile, supply kit preparation may require separate campaigns that address logistical barriers such as cost, storage, or transportation, particularly for low-income or physically limited populations. Together, these tailored, behavior-specific strategies can make hurricane preparedness messaging more effective and equitable across diverse communities.

### 5.1. Research significance, limitations, and future considerations.

The intellectual merit of this research lies in its contribution to the understanding of risk perception in the context of housing conditions during a disaster. By examining the relationship between housing physical characteristics and risk perception, the research identifies specific factors that influence how individuals perceive threat possibility and severity during hurricanes. This adds a nuanced understanding to the existing body of disaster studies and risk assessment. In addition, the research study explores how risk perception impacts individuals’ intentions to undertake protective actions. Although the correlations found are weak, these findings are important for developing more effective disaster preparedness strategies and public policy. Moreover, the use of a large, diverse sample from five cities in Florida enhances the robustness and generalizability of the findings. This methodological rigor adds to the credibility and reliability of the results.

Simultaneously, the broader impact of this research can be observed in several areas. First, by identifying specific housing conditions that influence risk perception, the research study can inform targeted interventions and educational campaigns to improve disaster preparedness among vulnerable populations. This can lead to better resource allocation and more effective community outreach programs [37,38]. Second, the findings can guide policymakers and urban planners in designing and implementing building codes and housing regulations that enhance safety and resilience against hurricanes. This can result in improved living conditions and reduced vulnerability for residents in hurricane-prone areas [39,40]. Third, understanding the weak correlation between risk perception and protective behaviors highlights the need for more effective communication strategies to bridge this gap. Educational programs can be tailored to address the specific perceptions and misconceptions that hinder protective actions, thereby increasing overall community resilience. Finally, this research study opens up new avenues for research on the interplay between housing conditions and disaster risk perception. It encourages further investigation into other potential factors that might influence protective behaviors, as well as the development of more comprehensive models to predict and enhance disaster preparedness.

On the other hand, our research has limitations and the first limitation is the sample size. We were able to collect 816 valid survey responses from five different cities in Florida, and that is a decent size for a convenience sample, and it meets the requirements for 95% confidence level and 5% margin of error if we considered the population of all cities together. Nevertheless, we weren’t able to collect enough survey responses to represent each city individually at the same confidence level and margin of error, except for Gainesville. Additionally, around 65% of the sample was from Gainesville alone, and that because Gainesville was more collaborative in sharing the survey compared to the other cities. Future studies should focus on collecting enough responses from each city, preferably with a 95% confidence level and 5% margin of error, as these are common for sample sizes in similar studies.

More than 90% of the survey responses came from cities in central and north Florida, and there was only one city from south Florida that participated in the survey (Miami). This is very important to be considered when generalizing any findings because cities in south Florida differ from the ones in central and north Florida in two main things. First is hurricane exposure, which can affect subjects risk perception and past hurricane experiences, and that could influence their intention to prepare for hurricane. Second is ethnicities and cultural differences. The dominating ethnicity in many of the cities in south Florida is Latino/Hispanic, while around half of the subjects in the survey were White. Different cultural back grounds can significantly affect how people look at and approach storm preparedness. The findings in this research study are more representative to central and north Florida, and so, future studies should consider collecting more data from south of Florida and compare it to the findings in this research study.

A notable limitation of this study is the geographic diversity of the selected cities, which may affect the generalizability of the findings to the state of Florida as a whole. While the inclusion of urban and inland cities such as Miami, Tampa, Gainesville, Jacksonville, and Tallahassee was intended to capture a wide range of demographic and environmental conditions, the lack of geographic proximity and shared climate features among them introduces potential variability that could mask localized trends. To address this limitation, future research could focus on more geographically cohesive regions—such as a single coastal zone or hurricane evacuation region—to allow for more context-specific analysis. This approach could strengthen the consistency of findings and provide more targeted recommendations for emergency management strategies within similar geographic and socio-economic settings. Additionally, conducting comparative studies between regions could also help identify how geographic factors mediate risk perception and preparedness behaviors.

Additionally, the female/male ration in the survey responses was around 62/35. This could have affected the analysis outcome because there are fundamental differences in the way different genders approach storm preparedness. Also, there were only 17 non-binary subjects out of the 816, Future studies should try to collect more responses and find different ways to engage non-binary individuals in such studies. At the same time, human behavior is generally very complex, and there could be many other constructs that influence people behavior during disasters [41]. Future studies should consider more behavioral constructs. Finally, the findings of this research study are specific to the context of Florida, even though they might be relevant to other hurricane-prone areas in the US, such as Texas, Alabama, and South Carolina. Similarly, the findings are specific to the context of hurricanes even though they might be relevant to states that are prone to other severe weather hazards, such as tornados and thunderstorms.

## 6. Conclusion

This research explored the intersection of housing conditions, risk perception, and hurricane preparedness behaviors across five cities in Florida, aiming to understand how individuals’ living environments shape their perceptions of hurricane threat and, subsequently, their intentions to take protective actions. Despite widespread assumptions about the role of risk perception in motivating preparedness, this study revealed that the relationship is far from straightforward. Findings showed that only two housing characteristics—required dwelling repairs and whether the home was on the ground floor—were significantly associated with variations in perceived risk, particularly for threat possibility. Other structural variables, such as building materials or floor level, did not exhibit strong associations. Furthermore, although perceived threat possibility and severity were expected to predict individuals’ intentions to prepare for an emergency supply kit, evacuation plan, or communication plan, logistic regression analysis showed that these perceptions were not statistically significant predictors. While some odds ratios suggested a positive relationship, their wide confidence intervals and high p-values undermined their reliability. These results align with other studies that have questioned the predictive power of risk perception in driving preparedness behaviors, particularly in the absence of an immediate threat. These insights have critical implications for emergency management. A static, one-size-fits-all communication strategy is unlikely to be effective across the entire hurricane season. Instead, preparedness campaigns should be dynamic, shifting from practical, non-threatening messaging during calm periods to urgency-driven appeals as storms approach. Moreover, bundling behaviors such as evacuation and communication planning into cohesive campaigns may enhance engagement, while separate, tailored strategies should address the unique barriers to supply kit preparation. This study contributes to a more refined understanding of how environmental, psychological, and behavioral factors interact in shaping hurricane preparedness. While risk perception alone may not consistently motivate action, context-sensitive interventions that consider housing vulnerabilities, behavioral patterns, and timing may hold greater promise for increasing resilience at the household and community levels.

## Supporting information

S1 Table Data Set (N=816). (XLSX)
